# The kinetics of inorganic phosphate excretion in the acidotic rabbit during intravenous phosphate loading: a pseudo-ruminant model

**DOI:** 10.1038/s41598-020-61069-0

**Published:** 2020-03-04

**Authors:** Patrick A. Walsh, Daniel J. O’Donovan

**Affiliations:** 1 Department of Physiology, School of Medicine, RCSI Bahrain, Manama, Kingdom of Bahrain; 2 Department of Physiology, National University of Ireland Galway, Galway, Ireland

**Keywords:** Kidney, Nephrology

## Abstract

The rabbit is a much-used experimental animal in renal tubule physiology studies. Although a monogastric mammal, the rabbit is a known hindgut fermenter. That ruminant species excrete inorganic phosphate (Pi) mainly through the digestive system while non-ruminants eliminate surplus phosphate primarily through the renal system are acknowledged facts. To understand phosphate homeostasis in the acidotic rabbit, anaesthetized animals were infused with hydrochloric acid, after which they underwent intravenous phosphate loading. Biofluids were collected during the infusion process for analysis. Plasma Pi increased (7.9 ± 1.7 mmoles.Litre^−1^ (N = 5) vs 2.2 ± 0.4 mmoles.Litre^−1^ (N = 10) pre-infusion, (p < 0.001)), while urinary phosphate excretion was also enhanced (74.4 ± 15.3 from a control value of 4.7 ± 3 µmol.min^−1^ (N = 9), pre-infusion, p < 0.001)) over an 82.5 minute Pi loading period. However, the fractional excretion of Pi (FePi) only increased from 14.2 ± 5.4% to a maximum of 61.7 ± 19% (N = 5) over the infusion period. Furthermore, the renal tubular maximum reabsorption rate of phosphate to glomerular filtration rate (TmPi/GFR) computed to 3.5 mmol.L^−1^, while a reading of 23.2 µmol.min^−1^.Kg.^0.75^ was obtained for the transport maximum for Pi (TmPi). The high reabsorptivity of the rabbit nephrons coupled with possibly a high secretory capacity of the salivary glands for Pi, may constitute a unique physiological mechanism that ensures the rabbit hindgut receives adequate phosphate to regulate caecal pH in favour of the resident metabolically - active microbiota. The handling of Pi by the rabbit is in keeping with the description of this animal as a monogastric, pseudo-ruminant herbivore.

## Introduction

The rabbit (*Oryctolagus cuniculus*) has an organ called the caecum that functions in a similar fashion to that of the rumen in a cow. However, unlike ruminants, this fermentation vessel in the rabbit is positioned not proximal to, but is located distal to the small intestine. The caecum in the rabbit has a capacity of about 10 times that of the stomach and about 40% of the total digestive tract^1^. A substantial portion of the rabbit’s nutrient digestion takes place in the caecum through the activity of a diverse population of autochthonous microorganisms, known collectively as the microbiota^2^. Through the practice of caecotrophy, the rabbit is able to retrieve the micronutrient rich soft faeces at night, directly from the anus, and reingest it for processing and absorption of its content in the small intestine. The practice of caecotrophy is reported to contribute about 83% more niacin, 100% more riboflavin, 165% more pantothenic acid, 42% more cyanocobalamin (vitamin B12), and100% more protein to the diet of a rabbit than is available without caecotrophy^1,2^. Because the rabbit does not regurgitate its food and chew the cud, but does rely on gut fermentation and caecotrophy for its nutritional wellbeing, this herbivorous species is sometimes referred to as a “pseudo-ruminant”.

The daily intake of phosphorous by a rabbit depends on its diet. In the wild, the rabbit obtains much of its phosphorous from consuming Lucerne (Alfalfa) and available vegetables. The phosphorous is released from the phytates in the food through the action of microbial phytases in the rabbit’s caecum. In the case of laboratory bred animals, the rabbits are provided with a pelleted diet that supplies, at a minimum, an amount of 1 g phosphorous per kilogram body weight as dicalcium phosphate^3^. In the case of commercial rabbit production units, the availability of phosphorous may be further enhanced by the addition of exogenous phytases to the formulated diet.

After absorption in the gut, phosphorous is transported across cell membranes, as inorganic phosphate (Pi), by means of a secondary active transport process, wherein it exists as the main intracellular anion. 80–85% of total body phosphate is located within bone, while ~15% is present within cells. Phosphate in plasma constitutes <1% of the total body stores. At the physiological pH of 7.4, phosphate exists in the plasma as a ratio of [HPO_4_^2−^]: [H_2_PO_4_^−^] ~ 4:1. While most of the Pi in the plasma is free, approximately 10% is protein bound and 5% is complexed with sodium, calcium, and magnesium. 90–95% of the plasma phosphate is ultrafilterable at the glomerulus. Maintenance of the plasma concentration of Pi within a relatively narrow range is essential for a number of key cellular activities, including energy metabolism, signal transduction, bone formation, nerve signalling, muscle contraction, and as a constituent of nucleic acids, phospholipids, and milk in lactating animals. The buffering roles of phosphate in extracellular fluid, in the urine, and in the digestive system of ruminant species are well known.

The regulation of plasma phosphate levels is governed by a set of complex activities taking place in a series of feedback loops, involving the parathyroid gland – gut - bone – kidney axis^4^. In respect of the renal handling of phosphate, the TmPi/GFR has proven to be clinically a very useful index. TmPi/GFR measures maximum renal tubular phosphate reabsorption in mass per unit volume of glomerular filtrate. In adult humans, the 95% reference range is 0.8–1.35 mmol.L^−1^ ^5,6^. Two main families of phosphate transporters facilitate the renal reabsorption of phosphate. These include the SLC34 family (NaPi-IIa & NaPi-IIc) and the SLC20 family (PiT-1& PiT-2), respectively^7,8^.

In addition to nutritional factors, a number of key regulators have been shown to play crucial roles in enabling the interplay between the organs regulating the plasma levels of phosphate. These include parathyroid hormone (PTH), calcitriol, and a number of phosphatonins, of which fibroblast growth factor-23 and α-klotho have been the best characterized. Klotho acts as a co-factor that is mandatory for FGF23 action^7,8^.

Phosphate loading studies have been carried out in a wide variety of species, including man^9^, the dog^10,11^, rodents^12^, the rabbit^13–15^, ovine^16^, and bovine animals^17^, respectively. In the course of calibrating the stimulatory effect of intravenous isotonic sodium phosphate on urinary acid – base parameters in the acidotic rabbit, it became clear to us^15^ that the kinetics of phosphate excretion in this herbivore is notably dissimilar to that which is characteristically found in monogastric animals. In this paper, we demonstrate that in the acidotic, hyperphosphataemic rabbit, at least two mechanisms are active in regulating plasma phosphate concentration. These include the excretory role of the rabbit kidney along with an additional, equally effective system, involving possibly the salivary secretion of phosphate into the digestive tract.

## Methods

### Ethical approval

The study was carried out at the Physiology Laboratories of the National University of Ireland Galway (NUIG) in Ireland. The animal experiments were performed in accordance with Directive 2010/63/EU, and ethics principles described by Grundy^18^. A permit to import the experimental animals from the United Kingdom was obtained from the Department of Agriculture (Veterinary Section), Kildare St., Dublin 2, Ireland. A license to undertake the animal experiments described in this paper, including ethical approval, was granted by the animal licensing committee at the Public Health Division of the Department of Health, Hawkins House, Dublin 2, Ireland.

### Animal husbandry

Specific Pathogen Free (SPF) male New Zealand White (NZW) rabbits were used in the study. The animals were 3–6 months old, and weighed 3.28 ± 0.54 Kg. The rabbits were obtained from a commercial supplier (Hylyne Rabbits Ltd, United Kingdom). They were housed individually in cages, in an environmentally controlled room (20 ± 1 °C, 35–60% relative humidity, and 12:12 light-dark cycle). The animals were allowed free access to water and rabbit chow (Bio resources unit, Trinity College Dublin, Ireland).

### Pre-operative preparation

On the day of the surgery, each animal (who had been fasted overnight) was pre-medicated with chlorpromazine hydrochloride (Largactil, Sanofi-Aventis: 2.5%, 25 mg.Kg^−1^), followed 30–45 minutes later with intravenous pentobarbital (Nembutal, Abbot Laboratories: 5%, 15 mg.Kg^−1^) through a marginal ear vein. The anaesthetized rabbit was placed in dorsal recumbency, on a small operating table, and secured in position using four limb-restraining robes. Rectal temperature was maintained throughout the surgery at 39 °C, using a homoeothermic blanket system with a flexible measuring thermistor probe. The depth of anaesthesia was monitored closely during experimentation by way of cardiorespiratory measurements and checking for the absence of the palpebral and pedal reflexes, respectively.

A 12 FG de Pezzer urethral catheter was placed in the urinary bladder as described previously^19^, to facilitate the collection of timed urinary specimens during the induction of acidosis and afterwards during phosphate loading. Control urinary samples were collected from each animal over a 15 – minute period, immediately preceding the commencement of the intravenous phosphate loading process at zero time. At the end of the study, the animals were sacrificed by anaesthetic overdose.

### Infusion of solutions

Infusions were made through the left jugular vein via an indwelling P.E. 50 cannula, which was connected to a loaded 60 ml syringe, positioned on a Harvard I.V. syringe pump (model 2681). The animals were infused with isotonic saline for two hours before being rendered metabolically acidotic, as described in previous studies^14,15^. The hydrochloric acid infusion was replaced by isotonic saline for 30 ± 5 minutes, to allow a steady-state metabolic acidosis to become established, prior to the initiation of phosphate loading via the intravenous infusion of 0.1 M sodium phosphate (pH 7.4) at 120 µmol.min^−1^.

### Measurement of phosphate

Control blood and urinary samples were taken from each acidotic animal prior to the commencement of phosphate loading. Plasma & urinary inorganic phosphate (Pi) concentrations were determined spectrophotometrically^20^.

### Data and statistical analysis

The data are presented as means ± SD, and statistical comparisons were performed using a Student’s paired *t* test, with statistical significance being taken as *P* ≤ 0.05. Statistical analysis was made using GraphPad Prism version 8.0 for Windows, (GraphPad Software, La Jolla California USA, www.graphpad.com.)

## Results

**Figure 1:**
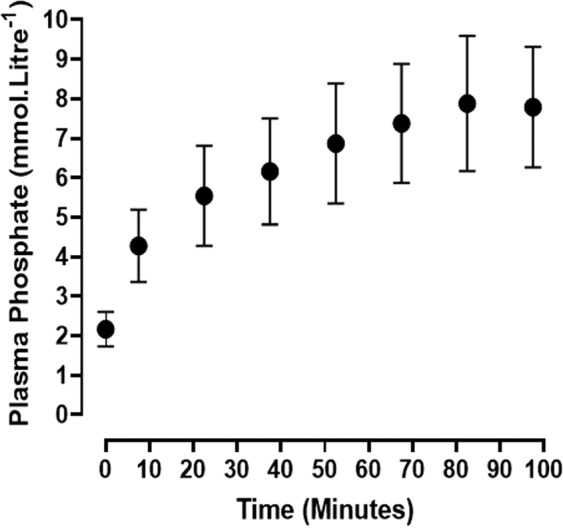
Plasma phosphate concentrations during the course of isotonic sodium phosphate (pH 7.4) infusion at 120 µmol.min^−1^. The results are plotted as the Means ± 1 S.D. (N = 6).

During the course of phosphate infusion, blood was sampled at the midpoint of each urinary collection period and harvested for plasma. Bioanalysis of the plasma samples showed that the phosphate concentration increased from a control value of 2.2 ± 0.4 mmol.Litre^−1^ (N = 10) to reach a maximum concentration of 7.9 ± 1.7 mmol.Litre^−1^ (N = 5), (p < 0.001) at 82.5 minutes from the time of commencement of sodium phosphate infusion (Fig. 1). The increase occurred in three discernible phases. An early dose-dependent increase over the initial 20–25 minutes of the infusion process was followed by a period during which the rate of increase in plasma Pi began to reduce, after which an acute steady state was reached between 82.5 and 97.5 minutes, despite continuous infusion of sodium phosphate buffer solution, pH 7.4.

**Figure 2:**
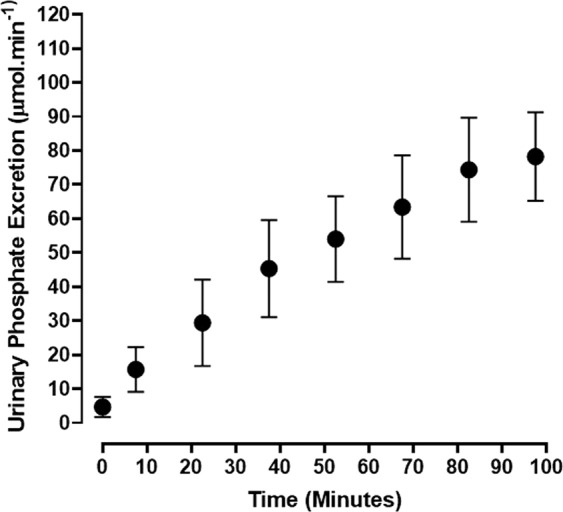
Urinary excretion rate of phosphate (Pi) during the course of phosphate infusion at 120 µmol.min^−1^. The results are presented as the Means ± 1 S.D. (N = 9).

**Figure 3:**
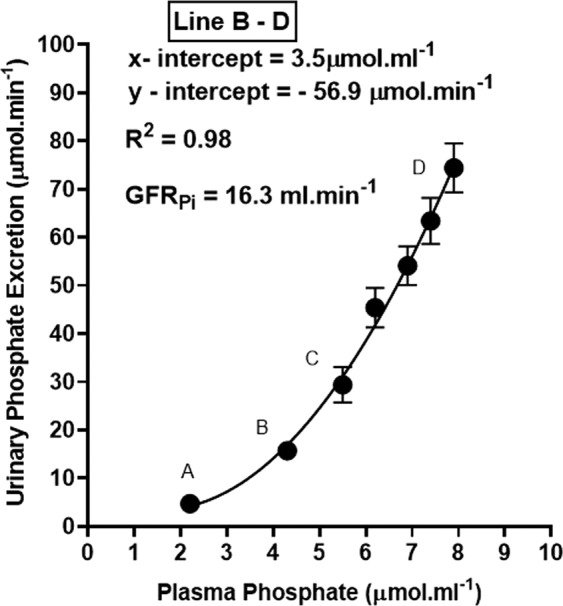
Plasma Pi concentration versus the urinary excretion rate of phosphate during the course of phosphate loading at 120 µmol.min^−1^. The values shown are the Means ± SEM.

Urine was collected at 15-minute intervals during the course of inorganic phosphate loading of the acidotic rabbits. Even though isotonic sodium phosphate was being intravenously infused at a rate of 120 µmol.min^−1^, the maximum urinary phosphate excretion rate that was attained over the 82.5 minutes of phosphate loading was 62% lower at 74.4 ± 15.3 from a control value of 4.7 ± 3.0 µmol.min^−1^, N = 9, p < 0.001) (Fig. 2). Kinetic analysis of the plasma and urinary phosphate data showed that the urinary excretion of phosphate increased in a linear fashion above the TmPi/GFR value of 3.5 mmol.L^−1^ (Fig. 3). This is very similar to what has been observed in such ruminants as the sheep^21^ and dairy cow^17^, but 3–4 fold greater than that found in man^5^.

Assuming a saturation kinetics model for phosphate reabsorption, a value of 16.3 ml.min^−1^ was calculated from the slope of the line for the glomerular filtration rate (GFR_Pi_), equating to an average GFR of 5 ml.min^−1^.kg^−1^ for the group of rabbits under study (Fig. 3). Taking into consideration the extracellular fluid volume expansion that would have taken place following the infusion of isotonic fluids over the experimental period, the glomerular filtration data reported here compare favourably with the inulin measurements of GFR obtained in the conscious rabbit by Michigoshi *et al*.^22^.

**Figure 4:**
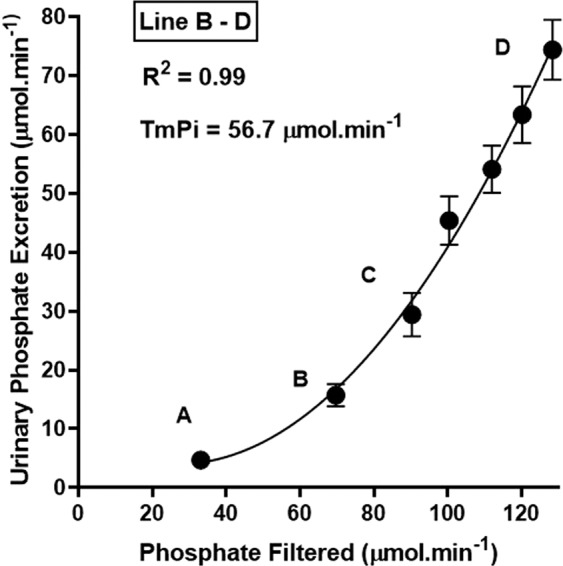
The rate of phosphate filtration versus the rate of urinary excretion of Pi during the course phosphate loading at 120 µmol.min^−1^. The values are shown as the Means ± SEM.

An evaluation of the filtered loads of phosphate reaching the nephrons over the experimental period showed that an average transport maximum for inorganic phosphate (TmPi) of 56.7 µmol.min^−1^ or 23.2 µmol.min^−1^.Kg.^0.75^ was reached within 20 minutes from the time of commencement of the Pi loading process (Fig. 4). The TmPi value for the rabbit compares very well with that which has been reported for such ruminants as sheep and cattle, but very different to what is found in man, dog, or rodent^21^. The renal fractional excretion of phosphate (FePi) increased from a control value of 14.2 ± 5.4% (N = 6) to reach a maximum of 61.7 ± 19% (N = 5) over the course of the infusion period. Furthermore, it is evident from the data (Table 1) that only after 80 minutes of phosphate loading did the rate of glomerular filtration of Pi approach the intravenous infusion rate of isotonic sodium phosphate.

**Table 1:** Renal phosphate excretion kinetics in the acidotic rabbit during infusion of isotonic sodium phosphate (pH 7.4) at 120 µmol.min^−1^.

Time (Minutes)	Pi Filtered (µmol.min^−1^)	Pi Excreted (µmol.min^−1^)	Fraction Pi Excreted FePi (%)	Pi Reabsorbed (µmol.min^−1^)
0	33.1 ± 7.8 (6)	4.7 ± 2.9 (11)	14.2 ± 5.4 (6)	27.3 ± 9.6 (5)
7.5	69.7 ± 15.0 (6)	15.7 ± 6.6 (12)	22.5 ± 10.8 (6)	50.4 ± 15.5 (6)
22.5	90.3 ± 20.7 (6)	29.4 ± 12.7 (12)	32.6 ± 16.7 (6)	63.1 ± 19.9 (6)
37.5	100.4 ± 21.9 (6)	45.4 ± 14.3 (12)	45.2 ± 18.1 (6)	51.5 ± 32.1 (6)
52.5	112 ± 24.8 (5)	54.1 ± 12.6 (10)	48.3 ± 18.7 (5)	52.7 ± 25.3 (5)
67.5	120.2 ± 24.5 (5)	63.4 ± 15.2 (10)	52.8 ± 19.9 (5)	52.2 ± 23.9 (5)
82.5	128.4 ± 27.9 (5)	74.4 ± 15.3 (9)	57.9 ± 21.6 (5)	42.1 ± 21.3 (4)
97.5	127 ± 24.9 (5)	78.3 ± 13.0 (7)	61.7 ± 19 (5)	36 ± 27.9 (2)

A GFR_Pi_ of 16.3 ml.min^−1^ was used to calculate the filtered Pi. The results are shown as the Means ± S.D.

The relatively low FePi profile (Table 1) coupled with a relatively constant plasma concentration of Pi, over the course of phosphate loading period, clearly provides evidence for the existence of an extrarenal pathway for Pi elimination in the experimental animals. This continuously active and very powerful inorganic phosphate scavenging mechanism alleviated the impact of both the parenterally administered and renal reabsorbed phosphate. Only in the last half of the phosphate-loading period, did renal elimination reach parity with the extrarenal route of phosphate disposal. We did not pursue to identify the nature of the extrarenal phosphate clearance mechanism(s), as it was outside the scope of the study at the time.

## Discussion

The handling of phosphate has been studied extensively in monogastric animals and ruminants. In such monogastric species as man, rodents, and the dog, extracellular phosphate balance is regulated principally through renal phosphate excretion^4,8,23^. However, in the case of ruminants such as the cow, sheep, and goat, phosphate homeostasis is executed primarily within the digestive tract, through the mechanisms of salivary secretion and faecal excretion^16,17,24^. On route to undergoing disposal via faecal excretion in the ruminant, it is thought that phosphate fulfills an important buffering function in the rumen, where microbial fermentative processes produce enormous quantities of volatile short chain fatty acids.

The data analytics of our study on the acidotic, hyperphosphataemic rabbit, would indicate that phosphate homeostasis in this species is consistent with a ruminant model. Unlike other monogastric species, it is evident that in the rabbit, an extrarenal route of Pi elimination is the predominant mechanism by which excess inorganic phosphate is removed from this animal’s body during the induced hyperphosphataemia. Analogous to other herbivores such as the sheep and goat, the rabbit may use its salivary glands as a phosphate-scavenging pathway to remove the toxic loads of phosphate from the blood plasma, which are ultimately excreted via the digestive tract. There may lie within the rabbit’s salivary glands a PTH independent phosphate sensing mechanism that operates to regulate plasma phosphate levels. Supporting this hypothesis, is the observation by other investigators that in both intact, and thyroparathyroidectomised sheep, there exists a linear relationship between acutely induced changes in plasma phosphate concentration, and the phosphate level in parotid gland produced saliva^16^.

In the rabbit, there are four pairs of salivary glands. In order of size, they include the parotids, mandibular, sublingual, and zygomatic glands, respectively. Their anatomy^1,25^ and histology^26,27^ have been well described in the literature. The primary formation of saliva is known to take place in the terminal portions of the salivary glands, where each of the epithelial cells, takes up interstitial fluid formed by local blood capillaries, processes it, and secretes the formed fluid, containing water, electrolytes, and macromolecules, in a unidirectional manner, from the basolateral region to the apical lumen of each acinus. From the lumen, it passes through the ductal system where it may undergo further modification on its way to being secreted into the mouth. The transcellular events, which play key roles in the formation of saliva within the epithelial cells of the acini, have been studied extensively^28,29^. In relation to phosphate, it has been shown that a Na^+^- coupled Pi transporter (NaPi-IIb (SLC34A2)), which utilizes the inwardly directed Na^+^ gradient to concentrate Pi inside the cells, facilitates the uptake of phosphate by the basolateral membranes of the epithelial cells. Inorganic phosphate travels down its electrochemical gradient, exiting the cell through its apical membrane via an unidentified mechanism. It has been shown that this electrogenic Pi transporter has a concentrating capacity of 10,000. Concentrations of phosphate in the parotid saliva of sheep, for example, have been shown to reach the enormously high values of 20–40 mmolar^28^.

The ultimate fate of the salivary gland scavenged phosphate along the rabbit’s lower digestive tract was not assessed in our studies for several reasons. Because the anaesthetized rabbits were in a dorsal recumbent position throughout the experiments, it would have been difficult to observe any changes in salivary flow. Secondly, the relatively short duration of the phosphate-loading period (1.75 hours) would not have allowed sufficient transit time for the salivary gland retrieved phosphate to arrive in the small intestine and caecum.

That the salivary glands are the extrarenal route of Pi elimination in the rabbit remains to be confirmed in future studies. Furthermore, it needs to be ascertained as to whether one or all four pairs of salivary glands have the capacity to secrete copious amounts of inorganic phosphate. It has been shown, for example, that the mandibular salivary glands in the rabbit secrete a continuous flow of saliva, while this role is fulfilled by the parotids in ruminants, and the sublingual glands in dogs and cats^30^.

Unlike humans, the dog, and rodent species, in which the kidney is the major site of phosphate excretion control, the rabbit kidneys play much more of a conservation role in withholding phosphate for extrarenal use. With very similar renal threshold (TmPi/GFR) and TmPi values to those found in sheep and cattle, it is clear that rabbit nephron reabsorptive kinetics for Pi resemble more those of ruminants than either of man, dog or rodent. Only in the last quarter of the phosphate loading process, when the filtered load of phosphate approached the rate of infusion of Pi, did the renal fractional excretion of phosphate reach parity with the extrarenal route of Pi elimination. Furthermore, the high reabsorptive capacity of the rabbit nephrons for Pi reduces the animal’s urinary titratable buffer capacity, and in so doing limits its ability to deal with an acidosis condition^15^.

Although three renal transporters (NaPi-IIa, NaPi-IIc, and PiT-2) have been identified, NaPi-IIa and NaPi-IIc account for the apical reabsorption of 80% of the filtered phosphate^7^. NaPi-IIa has been shown to be an electrogenic transporter (couples 3 Na^+^ to 1 Pi) and has a preference for HPO_4_^2−^, while NaPi-IIc is an electroneutral transporter, couples 2 Na^+^ to 1 Pi, and prefers the monovalent form of Pi (H_2_PO_4_^−^) as a substrate^29^. NaPi-IIa plays the most important role in the unidirectional and transcellular reabsorption of phosphate in the proximal tubules.

The abundance and activity of NaPi-IIa, and NaPi-IIc, transporters on the apical membranes of the proximal tubule cells is regulated by dietary Pi, PTH, 1,25 di-hyroxycholecalciferol, and FGF-23/klotho^7^. Hyperphosphataemia increases the secretion of PTH by the parathyroid glands and FGF23 by osteocytes, osteoblasts, and osteoclasts. The actions of PTH on the kidney occur within minutes, while the renal effects of FGF23 takes several hours to days^31^. While each of these two hormones cause down-regulation of the Na^+^- Pi co-transporters^32^, FGF23 has also been shown to increase the expression of 24-hydroxylase, an enzyme that renders 1,25-dihydroxycholecalciferol (1,25-(OH)_2_D_3_) (calcitriol) inactive, further inhibiting phosphate reabsorption^33^. Moreover, a low plasma level of calcitriol has also been shown to result in an increased salivary Pi concentration and secretion in ruminants^34^. The cumulative actions of PTH and FGF23 promote a phosphaturia. However, as in the hamster, the phosphaturic effect of PTH has been shown to be reduced in rabbit nephrons relative to the impact of this hormone on rodent renal tubules^35,36^.

In an evaluation of the impact of parenteral phosphate feeding on phosphate metabolism in the experimental rabbit, it is important to be aware that a number of factors, independent of the phosphate loading process, can influence the level of background phosphaturia in these animals. In the context of the study described in this paper, these would have included diet and respective blood acid-base status of the animals at the time of phosphate administration.

Previous investigators^37^ have established that the proportion of phosphate excreted in the urine can vary depending on the acid/alkali content of their diet. For example, rabbits fed carrots (alkaline diet) excreted 25% of the ingested phosphate in the urine, while those fed a mixed diet of carrots and oats showed a 50% excretion as urinary phosphate, and finally those given oats exclusively (acidic diet) demonstrated a 100% excretion as urinary phosphate. The rabbits in this study were fed a commercial pelleted diet, formulated to the dietary specifications of an herbivore, as described in the husbandry section.

With respect to blood acid base status, animal studies have revealed that metabolic acidosis can promote a phosphaturia by at least three independent mechanisms. The first involves an acidosis-mediated inhibition of the glycolytic rate^38^, decreasing the cellular uptake of phosphate. Secondly, to enhance urinary net acid excretion, increasing amounts of phosphate are absorbed from the intestine and released from bone into the bloodstream, leading to an amplification of the urinary titratable acid excretion^39,40^. Thirdly, studies have shown that while PiT1 & PiT2 transporters are increased over 7 days during metabolic acidosis, there is a significant inhibition of NaPi-IIa transporter activity, leading to a decrease in the renal uptake of phosphate, further potentiating the phosphaturia. A modulation of the trafficking of the transporter protein to the apical cell membrane, the reduced availability of divalent HPO_4_^2−^ at urinary pH ≤ 6.0, and a decreased synthesis of NaPi-IIa protein have been collectively shown to account for the inhibited Na^+^/Pi co-transporter activity observed in metabolic acidosis^41^. In complete contrast, it has been shown that metabolic acidosis increases NaPi-IIb abundance in the ileum of the rat^42^, the physiological purpose of which is to facilitate an increased reabsorption of phosphate to buffer the extracellular acid load, in addition to minimizing the excessive liberation of phosphate from bone. In respect of the acidotic, hyperhophataemic rabbits in this study, it would be interesting to ascertain if salivary gland NaPi-IIb is affected in a similar fashion.

Phosphate, calcium, and magnesium metabolism are very closely interconnected in many monogastric animals, as similar regulating hormones govern their activities. However, in the rabbit, whereas the digestive system appears to be the primary route for phosphate excretion, urine is the major route for calcium and magnesium excretion^43,44^. In contrast to the intestinal control of Pi uptake by 1,25-(OH)_2_ D_3_, rabbit gut calcium absorption is independent of calcitriol regulation. Rather, a balance between dietary intake and urinary excretion determines the serum total calcium level in this species. Hence, a high intake of calcium is responsible for rabbit urine having a thick creamy appearance. High plasma citrate concentrations facilitate the urinary excretion of copious quantities of ultrafilterable calcium by this species^1^.

It is clear that future research studies are required to characterize the unique kinetic properties of the transporters responsible for phosphate reabsorption in the rabbit kidney, in addition to determining what hormonal and other factors might influence their respective activities during hyperphosphataemia. The extrarenal mechanisms of plasma phosphate regulation that appear to operate in the rabbit undoubtedly warrant further investigation. In this regard, the secretory role of the salivary glands in excreting inorganic phosphate into the digestive tract has to be investigated at the outset. The inter-relationship between the parathyroid and salivary glands in the homeostatic regulation of plasma phosphate levels in the rabbit requires a detailed appraisal, including identifying the location and biochemical nature of a plasma phosphate sensing mechanism. One might postulate that an avid reclamation and secretion of phosphate by the salivary glands, coupled with a ruminant–like kidney phosphate kinetics model, may make it possible for the rabbit caecum to harvest adequate amounts of inorganic phosphate to regulate the pH therein, and in so doing, prevent a transfaunation of the resident microbiota taking place^45^. The findings of this study confirm the description of the rabbit as a monogastric, pseudo-ruminant herbivore.
